# Supplementary material for: Sonification as a reliable alternative to conventional visual surgical navigation
Source: Sci Rep. 2023 Apr 12;13:5930. doi: 10.1038/s41598-023-32778-z (PMC10097653; doi:10.1038/s41598-023-32778-z)
Supplement: Supplementary file 3 — Supplementary Information 3. [file 41598_2023_32778_MOESM3_ESM.docx]

**Demonstration of Four DOF Sonification Model**

The video shows the four DOF sonification model for surgical tool navigation in pedicle screw placement. The model comprises two interactive phases, namely, entry-point and angle, and indicates the moment when the tool is at the target. The last part of the video briefly shows the experiment conducted on a phantom.
